# 2546: Mobile use patterns among low-income parents and teens enrolled in outpatient substance abuse treatment

**DOI:** 10.1017/cts.2017.153

**Published:** 2018-05-10

**Authors:** Stacy Ryan, Lindsay L. Lange, Donald M. Dougherty

**Affiliations:** University of Texas Health Science Center, San Antonio, TX, USA

## Abstract

OBJECTIVES/SPECIFIC AIMS: This study sought to determine the accessibility, utilization, and preference for mobile phone use among a marginalized population of teens enrolled in an adolescent substance abuse treatment program and their parents. Specific study aims were to: (1) characterize mobile phone use, (2) assess the accessibility and reliability of mobile phone usage, (3) determine specific barriers to mobile phone use, and (4) examine parent and teen perceptions of the utility of integrating communication technology in substance use treatment. METHODS/STUDY POPULATION: In total, 103 (78.6% female; 75.7% Hispanic) parents of teens participating in an outpatient substance abuse treatment program with an average age of 42.60 (SD=9.28) participated in our study. Upon enrollment in a substance abuse treatment program between October 2014 and July 2016, parents completed a technology use survey as part of program development and a chart review of clinic outbound calls to parent mobile phones was completed to evaluate reliability of parent mobile phone access throughout treatment. Survey collection among teens is ongoing. Study population information for teens will be presented at the conference. RESULTS/ANTICIPATED RESULTS: The vast majority of parents owned a cell phone and used it as their primary phone (97.1%); 83% of parents owned smart phones in particular, with the majority being Android phones (68.7%). Parents were more likely to have pay-as-you-go (41.4%) and yearly (32.3%) contracts, and only 15% of the sample endorsed changing their phone number more than once in the past year (64%=never; 21%=once). Parents reported using several of the phone features: text (97%), email (76%), pictures (93%), and accessing the internet (92%); 92% reported they did not have a texting limit; and the most popular use of the mobile phone was to send and receive text messages (58.6%), followed by accessing the internet (19.2%). During the course of a 10-week treatment program, the clinic made 2776 confirmation phone calls to parents who completed surveys. Report of accessibility matched the clinic’s ability to reach parents. Of the 2776 calls, 97.2% were made to the original number provided, which was in service. Only 2.7% were determined to be disconnected, with the median number of days for disconnected service being 2 days with no voice and no texting capabilities (range=14) and 2 days with no voice, but with texting capabilities (range=28). In terms of parent perceptions of the utility of integrating communication technology in substance use treatment, 91% of parents reported they would be receptive to receiving text messages with parenting tips as aftercare support. Preferred content areas included: strategies for monitoring teen substance use (56%), strategies for using consequences (62%), suggestions for encouraging positive activities (62%), and ways to improve parent-child communication (63%). Accessibility, utilization, and preference for mobile phone use in a treatment program among teen respondents will be presented at the conference. DISCUSSION/SIGNIFICANCE OF IMPACT: This study characterized both subjective and objective mobile phone accessibility and usability among teens participating in an adolescent substance abuse treatment program and their parents. This study also provides information on teen and parent perceptions of using mobile phones during the aftercare period and ratings of acceptable messages following treatment. This data will help researchers design mobile-based interventions both during and after treatment, which is the future direction of our research group.

